# Immune implication of FAM83D gene in hepatocellular carcinoma

**DOI:** 10.1080/21655979.2021.1950260

**Published:** 2021-07-24

**Authors:** Tao Meng, Zhong Tong, Ming-Ya Yang, Yan Zhang, Yu Liu, Zhen-Zhen Wang, Li-Xin Zhu, Jin Wu

**Affiliations:** a Department of General Surgery and Centre Laboratory, The First Affiliated Hospital of Anhui Medical University, Hefei, China; b Department of General Surgery, Hefei City First People’s Hospital, Hefei, China

**Keywords:** FAM83D, immune cells, HCC, prognosis, nomogram

## Abstract

FAM83D has been demonstrated to contribute to tumorigenesis. However, its immune effects in hepatocellular carcinoma (HCC) have not been reported. This study aimed to identify the immune role of FAM83D in HCC. FAM83D was over-expressed in HCC and contributed to poor prognosis according to the results of data analysis based on The Cancer Genome Atlas (TCGA). Afterward, the levels of immune cells infiltration were found to be correlated with the expression level of FAM83D in HCC. Through TISIDB and cBioPortal network tools, a total of 82 FAM83D-associated genes were screened out, including 12 immunoinhibitors, 20 immunostimulators and 50 tightly co-expressed genes. TCGA cohort was divided into train set and test set on the basis of the proportion of 7:3. According to FAM83D-associated immunomodulators, a four gene predicted model was established using train set via the Cox regression analysis. Survival analysis demonstrated that the overall survival (OS) of high-risk HCC patients was poor compared with the patients in low-risk group. The reliability and predicted power of the risk-score model were identified by a receiver operating characteristic (ROC) curve. A risk-score based nomogram as well as a calibration curve, which were created could be used to anticipate patient’s 1-year, 3-year and 5-year survival probabilities. The test set was used to validate these results. Our findings showed that the FAM83D gene was related with HCC immunity. The immune marker chosen could be a promising biomarker for HCC prognosis.

## Graphical abstract

**Figure:**
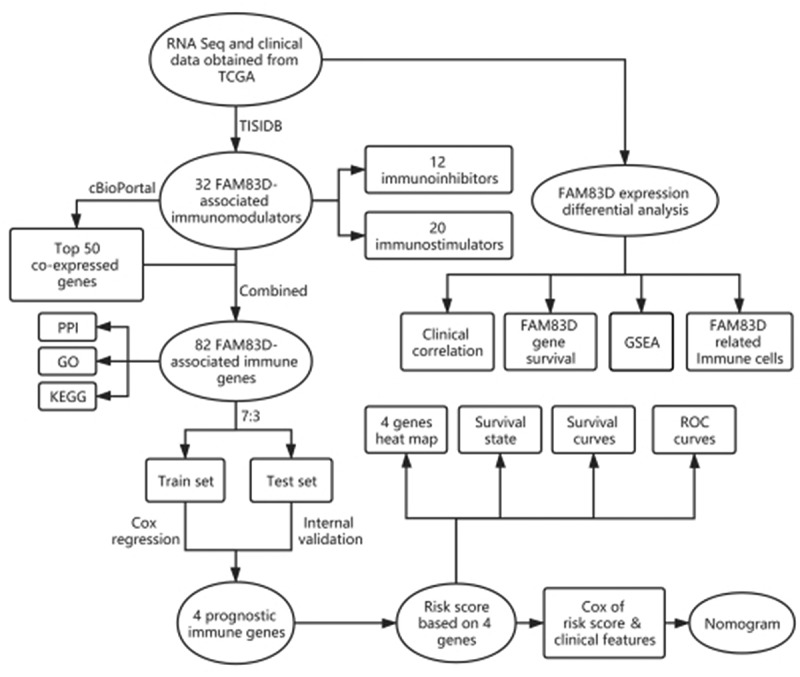

## 1. Introduction

Hepatocellular carcinoma (HCC), which constitutes 75%-85% of primary liver cancers, is a major health problem worldwide primarily because of the transmission of hepatitis B and C virus infection [1]. Many researches have identified multiple key genes and pathways related to the occurrence of HCC [2,3]. Although immunotherapy is a promising alternative therapy, only a part of HCC patients benefits from immunotherapy, which may be due to the limited effective targets [4–6]. Prognostic immune biomarkers can help identify subgroups of immunotherapy responses. Studies have reported that tumor-infiltrating leukocytes are correlated to tumor prognosis [7–9]. Therefore, the molecular pattern of the micro-environment within HCC immunity needs to be further explored. It is necessary to comprehensively understand the regulatory mechanisms of HCC immunology to ensure the success of immunotherapy.

FAM83D has been reported to be an oncogene in many cancers [10–12]. In HCC, studies have reported that FAM83D overexpression is associated with gender, AJCC stage, tumor recurrence and survival [13], and also related to recurrence after liver transplantation [14]. However, the immune role of FAM83D in HCC has not yet been found.

In this study, data contained mRNA expression and clinical features from The Cancer Genome Atlas (TCGA) Liver cohort were adopted to analyze the correlation between FAM83D gene, HCC immune micro-environment, and patients clinical parameters. Next, the FAM83D-associated immunomodulators and co-expressed immune genes were identified via TISIDB and cBioPortal network tools. Finally, the key gene signature based FAM83D-associated immune genes were generated by Cox regression to construct a prediction model, in addition, the key gene signature and clinical features were combined to construct a nomogram.

## 2. Material and Methods

### 2.1 Data harvesting

The mRNA profile and clinical data was obtained from TCGA official website (http://portal.gdc.cancer.gov/repository; updated 18 December 2020), including 374 tumor samples and 50 normal samples (FPKM format). To further process RNA expression data, the ‘limma’ package was used for R software [15].

### 2.2 Relationship between FAM83D and clinical characteristics

The differential pattern of FAM83D expression was analyzed in TCGA between tumor and adjacent tissues with ‘limma’ packages [15]. Patients with HCC were separated into two groups with high and low FAM83D expression, the survival curves were created by the Kaplan Meier analysis [16]. Kaplan Meier Plotter network tool was adopted to verify the survival difference (https://kmplot.com/analysis/). In addition, the correlation was analyzed between the expression level of FAM83D gene and the tumor stage, grade, age, gender and TMN classification of HCC.

### 2.3 Gene set enrichment analysis

All HCC patients in TCGA LIHC dataset were separated into high and low group regarding to the expression of FAM83D gene. Gene set enrichment analysis was utilized to analyze the signal paths that were significantly correlated to the expression level of FAM83D. GSEA evaluates gene expression pattern across the genome at a level of the gene set, rather than focusing on a small number of mostly altered genes [17].

### 2.4 Tumor infiltrating immune cells with TCGA LIHC RNA-Seq

Similar to previous research [18], to classify and evaluate 22 different kinds of immune cells in tumor samples, containing seven different types of T cells, naive and memory B cells, plasma cells, NK cells, and myeloid subsets, the Cell type Identification by Estimating Relative Subsets of RNA Transcripts (CIBERSORT) were adopted (https://cibersort.stanford.edu/) [19]. The TCGA LIHC data (fpkm format) was transformed into the TPM format to improve the comparability of the samples [20]. With CIBERSORT L22 as the benchmark, RNA-Seq data from TCGA LIHC was analyzed with the CIBERSORT R script obtained from the CIBERSORT website. For the deconvolution of each case, an empirical P-value was calculated using Monte Carlo sampling [21]. Samples were included with P < 0.05.

### 2.5 Immunomodulators

The TISIDB website was used to obtain tumor infiltrating lymphocytes (TILs) and immunomodulators that were related to FAM83D in order to clarify the tumor-immune system interaction in HCC (http://cis.hku.hk/TISIDB/) [22]. TILs and immunomodulators including immunoinhibitors and immunostimulators for gene expression that were significantly correlated with FAM83D were selected (Spearman correlation test, P < 0.05). Subsequently, the FAM83D-related immunomodulators were uploaded to cBioPortal (www.cbioportal.org) [23]. Based on the mRNA expression matrix of tumor tissues from HCC patients, the top 50 co-expressed genes were detected and identified. The seized proteins were used to analyze protein interaction with STRING network tool (https://string-db.org/) [24], and performed GO and KEGG analysis (http://consensuspathdb.org/) [25].

### 2.6 Survival analysis

The TCGA cohort was separated into train set and test set with the proportion of 7:3. Test set was used for internal validation. To screen out prognostic-related immune genes, univariate and multivariate COX analysis were utilized to analyze FAM83D-related immune genes in train set. Risk score was calculated with coefficients: risk score = β1x1 + β2x2 + … + βixi. Xi represented each gene’s expression level, and βi was the every gene’ coefficient obtained from the Cox analysis. The survival curve was adopted to study the relationship between risk-score of immune-related genes and the OS in HCC. The time-dependent receiver operating characteristic (ROC) curves were utilized to assess the predictive power of the immune-related genes signature.

### 2.7 Construction of nomogram

Nomograms are widely used in cancer prognosis to estimate the probability of a single event, such as death or recurrence, tailored to the characteristics of a single patient [26]. In this study, the parameters and risk scores of the patients were combined to create a nomogram that was used to assess the prognosis. The nomogram was created for R software via the rms package. The bootstrap method was utilized in conjunction with a calibration curve (1,000 replicates) to visualize the difference between predicted and actual probabilities. The forecasting precision of a nomogram was measured using the concordance index (C-index).

### 2.8 Internal validation

The TCGA test set was used for internal validation. Every HCC sample in the validation datasets had its risk-score estimated by the same formula based on the risk-score of prognostic signatures. Then the HCC patients were divided into high and low group in accordance with the risk-score. To evaluate the model’s predicted power, survival curves and ROC curves, were used.

### 2.9 Statistics

All statistical analysis procedures were carried out with R version 4.0.3. For the GSEA set enrichment analysis, GSEA software was used (GSEA Desktop Application v4.1, Broad Institute, Inc, Cambridge, MA, USA). Wilcoxon rank sum test was used to analyze the different expression of FAM83D, Kruskal–Wallis H analysis was adopted to explore the association between FAM83D and clinical features, univariate and multivariate COX regression analysis were adopted to analyze prognosis-related genes. *P* < 0.05 was considered statistically significant.

## 3. Results

In this study, we first studied the carcinogenic effects of FAM83D in HCC. Then we analyzed the relationship between FAM83D gene and immune cells in HCC and discussed the association with the immune micro-environment. Finally, through the online network of TISIDB and cBioPortal, FAM83D-related immunomodulators and immune genes were identified, and a prediction model was established to identify the key prognostic immune genes.

### 3.1 FAM83D was over-expressed in HCC and predicted poor prognosis

**Figure 1.**
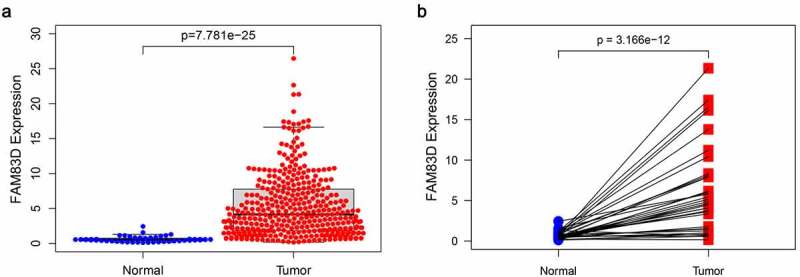
The expression pattern of FAM83D in TCGA. (a) differential analysis of FAM83D. (b) Paired differences in TCGA

**Figure 2.**
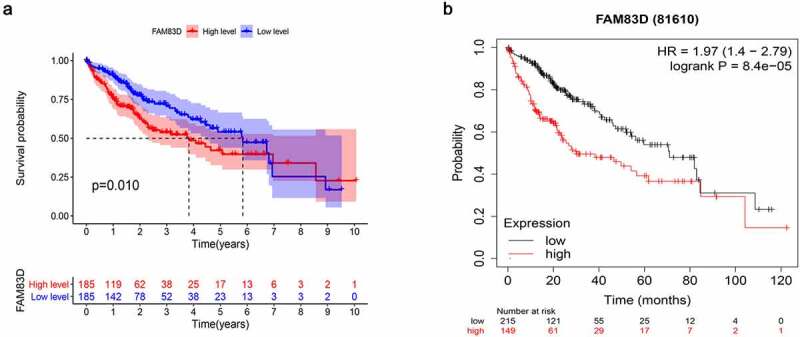
FAM83D expression associated survival analysis. (a) TCGA. (b) Kaplan meier plotter

FAM83D expression in tumors was found to be significantly higher than that of adjacent tissues according to differential analysis between 374 tumor and 50 normal tissues in TCGA RNA-Seq dataset (Figure 1a-b). The prognosis of patients for high FAM83D expression was worse than low expression group in TCGA datasets (Figure 2a) and Kaplan Meier Plotter (Figure 2b).

### 3.2 Relationship between FAM83D and clinical characteristics

**Figure 3.**
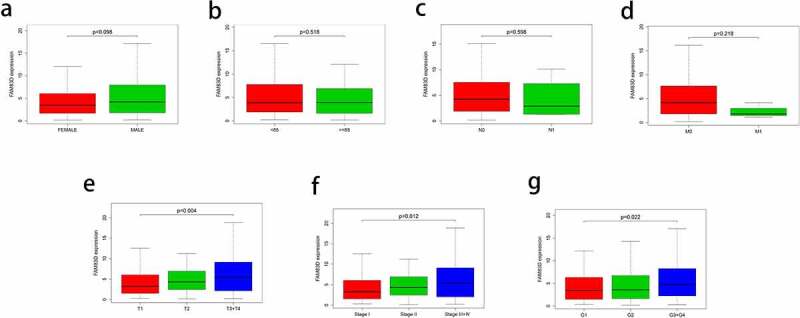
Association of FAM83D expression with clinical features. (a) Gender. (b) Age. (c) Lymphatic metastasis. (d) Distant metastasis. (e) T classification. (f) AJCC stage. (g) Grade

In the TCGA LIHC data set, Kruskal–Wallis H analysis was used to investigate the correlation between FAM83D and clinical features. The results showed that the expression level of FAM83D was significantly associated with T classification, AJCC stage and histologic grade (*P* = 0.004, 0.012 and 0.022, respectively. Figure 3). Logistic regression analysis showed that the expression level of FAM83D gene was also positively related to T classification, AJCC stage and histologic grade, but had no obvious correlation with variables such as age and gender. The expression level of FAM83D in AJCC stage 1, T1 and grade 1 group were significantly lower than that in late stage, late T classification and late grade. (*P* = 0.04303, 0.01235 and 0.02046, respectively, Table 1); The results of univariate COX regression analysis showed that the expression level of FAM83D, stage, T classification and N classification were significantly related to the overall survival of HCC patients (*P* = 6.44E-07, 3.83E-07, 0.02089 and 8.30E-05, respectively, Table 2). The results of multivariate COX regression analysis suggested that the expression level of FAM83D may be an independent risk factor for the prognosis of HCC patients (HR = 1.07, *P* = 0.003, Table 2).

**Table 1.** Logistic regression of FAM83D expression and clinical pathological characteristics

Clinical characteristics	Total (N)	Odds ratio for high FAM83D expression	95%CI	*P*-value
Stage classification (II vs I)	257	1.354	0.778–2.363	0.28342
(III +IV vs I)	261	1.792	1.022–3.172	**0.04303**
T classification (T2 vs T1)	275	1.566	0.950–2.593	0.07936
(T3+ T4 vs T1)	274	1.911	1.154–3.191	**0.01235**
Gender (male vs female)	321	1.532	0.955–2.473	0.07835
Grade classification (G2 vs G1)	232	1.122	0.610–2.086	0.71344
(G3+ G4 vs G1)	189	2.126	1.129–4.060	**0.02046**
N classification (N1 vs N0)	233	1.009	0.119–8.524	0.99309
Age (65 vs <65)	320	1.027	0.654–1.612	0.90851

**Table 2.** Univariate and multivariate analysis of the relationship between FAM83D expression and overall survival among HCC patients

Parameter	Univariate analysis			Multivariate analysis		
	HR	95%CI	*P*	HR	95%CI	*P*
Age	1.007	0.989–1.025	0.47951	1.008	0.989–1.028	0.40970
Gender	0.778	0.487–1.244	0.29470	0.957	0.569–1.611	0.86910
Grade	1.013	0.743–1.380	0.93439	1.076	0.773–1.498	0.66502
Stage	1.879	1.466–2.408	**6.44E-07**	1.000	0.379–2.638	0.99948
T	1.816	1.443–2.287	**3.83E-07**	1.659	0.690–3.990	0.25824
N	3.924	1.230–12.519	**0.02089**	1.856	0.483–7.136	0.36796
M	2.070	0.506–8.471	0.31164	2.542	0.424–15.238	0.30712
FAM83D	1.087	1.043–1.133	**8.30E-05**	1.070	1.023–1.119	**0.00307**

### 3.3 Gene set enrichment analysis

**Figure 4.**
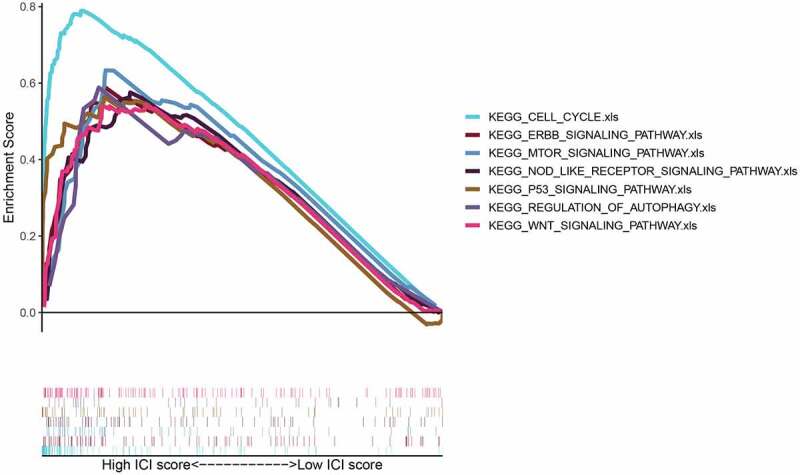
Enrichment plots from gene set enrichment analysis (GSEA)

To explore the up-regulated FAM83D expression associated pathways in HCC, GSEA analysis was used to study the datasets of tumor samples collected from TCGA. We found that elevated FAM83D participated in several paths, such as cell cycle, p53 signaling pathway, mTOR signaling, regulation of autophagy and Wnt signal pathway, which were involved in cell proliferation and immunity (Figure 4).

### 3.4 Infiltration of immune cells in HCC and normal samples

The CIBERSORT method was used to extract and process the TCGA RNA-Seq profile, and the pattern of immune cells was systematically described. By including samples with *P* < 0.05, an overview of the immune cell infiltration of HCC matrix was shown in Supplementary Figure 1a and Supplementary Figure 1d. The proportion of B cells naive, macrophages M0 and macrophages M1 were significantly increased when contrasted to healthy tissues, while B cells memory, T cells CD8 and T cells CD4 memory resting, monocytes, mast cells resting and eosinophils in HCC were reduced (Supplementary Figure 1b). The correlation between different immune cell subgroups was shown in Supplementary Figure 1c, indicating that there were different patterns of immune infiltration in HCC. In addition, the infiltration level of CD8^+^ T cells and CD4^+^ T cells as well as the expression level of FAM83D were significantly correlated with HCC survival (Supplementary Figure 2).

### 3.5 Relationship between FAM83D and immune cells

**Figure 5.**
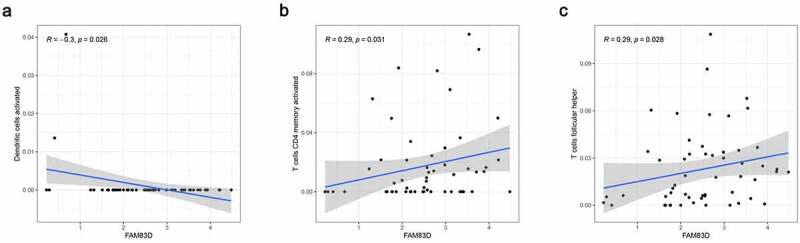
Associations between FAM83D and infiltrating immune cells in TCGA LIHC cohort

**Figure 6.**
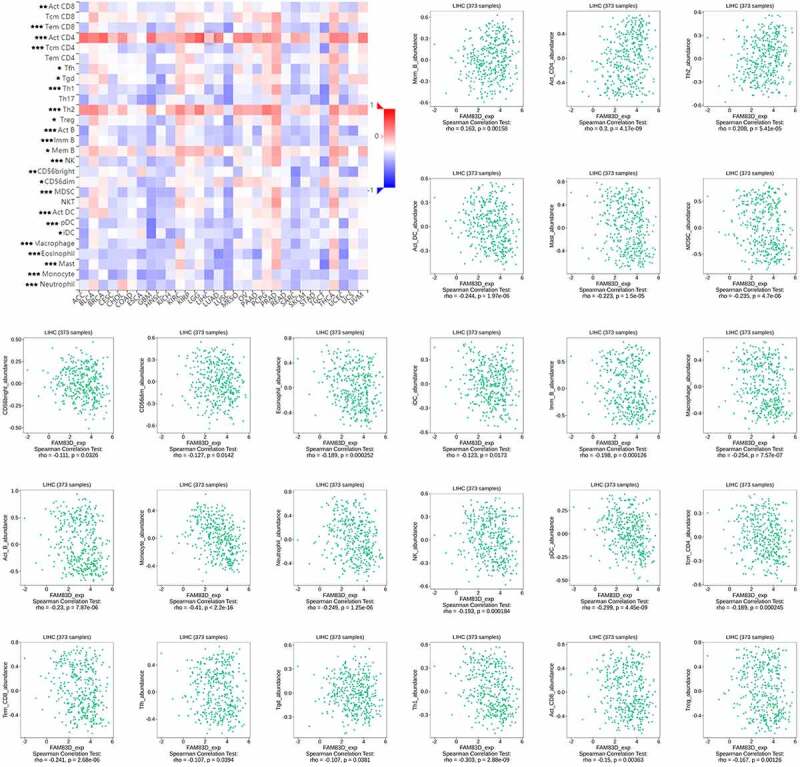
Association between FAM83D expression and immune cell infiltration levels via TISIDB network tool. The black asterisks in the correlation heatmap indicated immune cell types significantly associated with FAM83D expression levels in TCGA. *P < 0.05; **P < 0.01, ***P < 0.005

**Figure 7.**
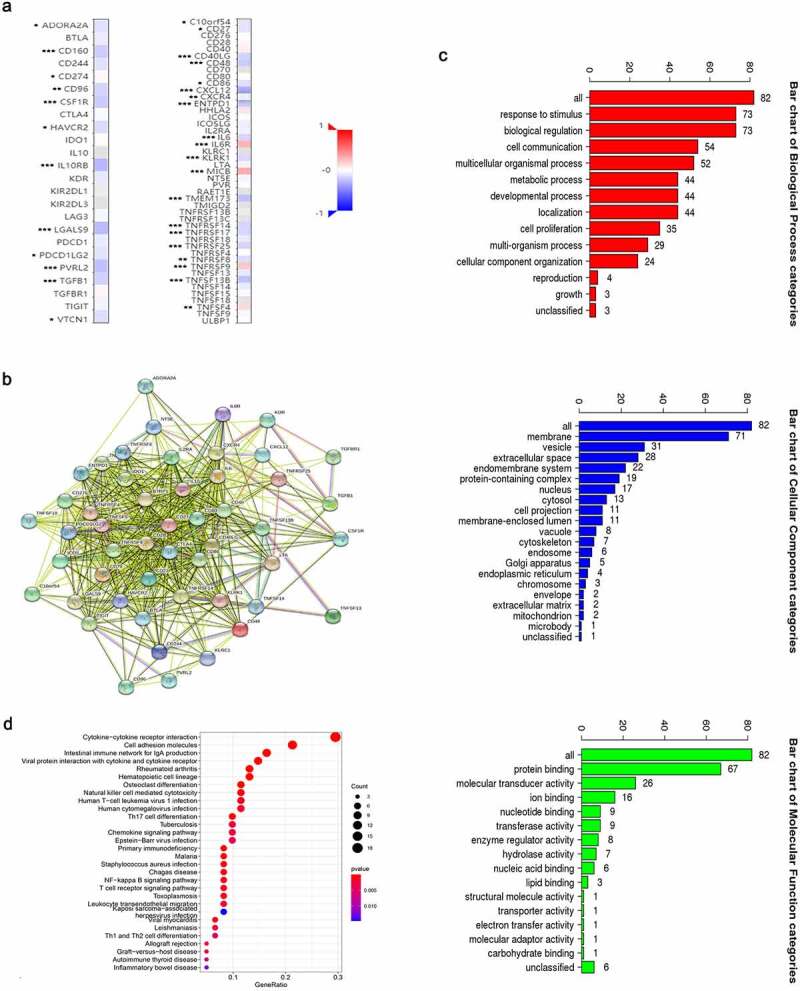
Identification and analysis of immunomodulators associated with the FAM83D gene. (a) The heatmaps of correlation between the immunoinhibitors (left panel), immunostimulators (right panel) and the FAM83D gene in LIHC. (b) Protein–protein network of 32 FAM83D-associated immunomodulators and 50 closely related genes in LIHC, produced by the STRING online server. (c) GO annotation of 32 FAM83D-associated immunomodulators and 50 closely connected genes in LIHC. (d) KEGG pathway analysis of the above mentioned 82 genes

We discovered through R software that the level of immune infiltration varied with the FAM83D expression in HCC, including DC cells, CD4^+^ T cells and T cells follicular helper (Figure 5). Moreover, some immune cell subsets in HCC that were positively or negatively correlated with FAM83D expression (Figure 6). Then we studied the possible immune response signaling pathway mediated by FAM83D. Through TISIDB, 32 immunomodulators related to FAM83D were identified, including 12 immunoinhibitors (ADORA2A, CD96, CD160, CD24, CSF1R, HAVCR2, IL10RB, LGALS9, PDCD1LG2, NECTIN2, TGFB1 and VTCN1) and 20 immunestimulators (VSIR, CD27, CD40LG, CD48, CD86, CXCL12, CXCR4, ENTPD1, IL6, IL6R, KLRK1, MICB, TMEM173, TNFRSF8, TNFRSF9, TNFRSF14, TNFRSF17, TNFRSF25, TNFSF4 and TNFSF13B) (Figure 7a). Subsequently, we selected the top 50 genes strongly tied to these 32 immunomodulators by using cBioPortal for Cancer Genomics (Figure 7b). GO was adopted to annotate these genes and KEGG analysis suggested that these genes were involved in the processes of immune signaling (Figure 7c-d).

### 3.6 Prognostic significance of FAM83D-related immunomodulators

**Figure 8.**
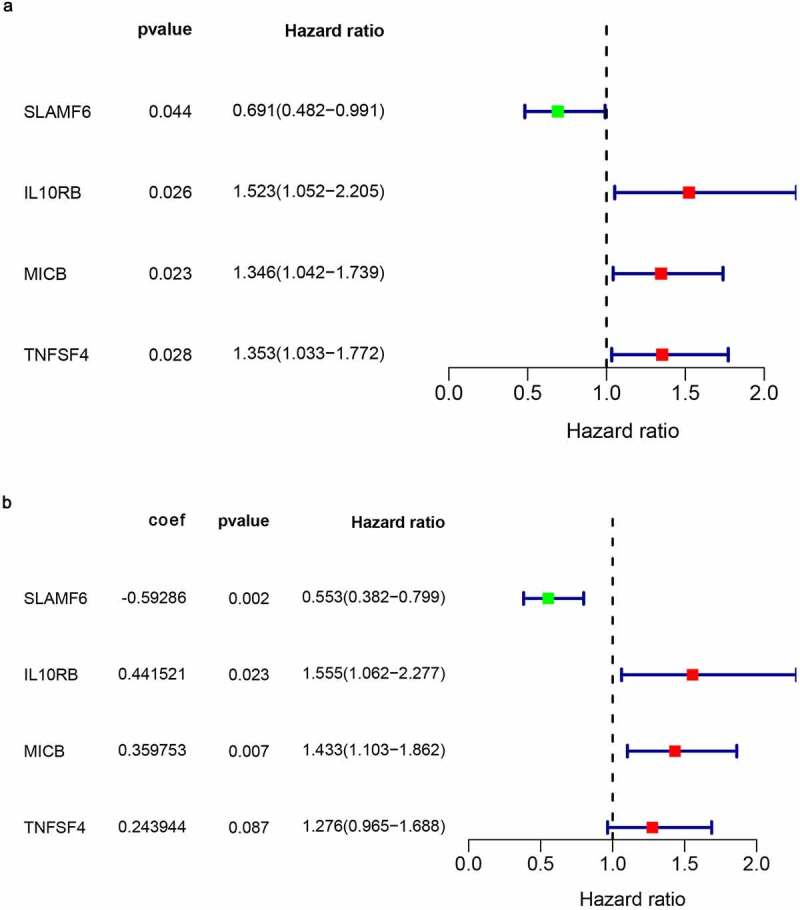
The development of prognostic gene signatures based on 82 FAM83D-associated immunomodulators. (a) Univariate COX regression of FAM83D-associated immunomodulators. (b) The hazard ratios and coefficient of FAM83D-associated prognostic genes of multivariate COX regression

**Figure 9.**
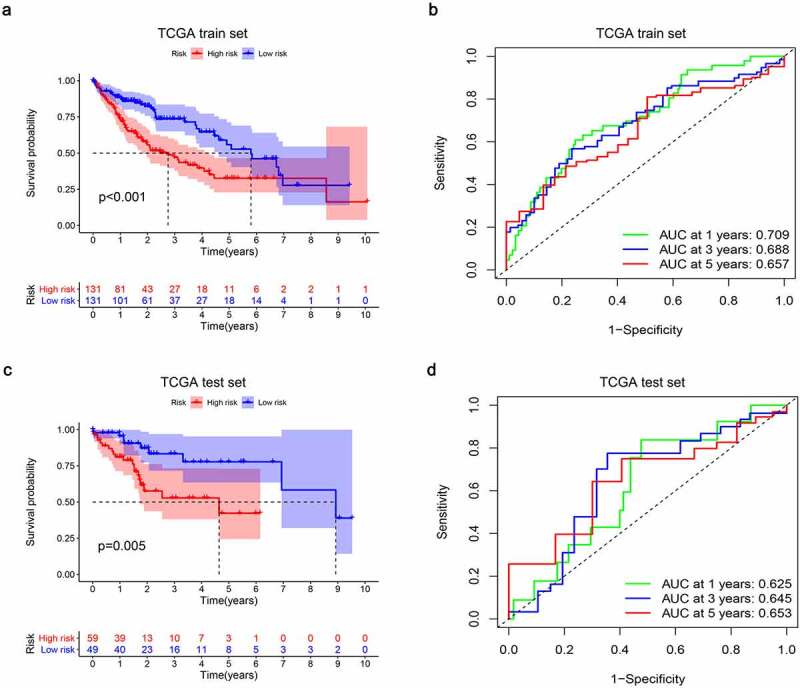
Establishment and internal validation of prognostic model based on risk-score of FAM83D-associated prognostic immunomodulators. (a) Survival curves of the high and low risk group of the TCGA train set. (b) Time-dependent ROC curves of FAM83D-associated prognostic model of the TCGA train set. (c) Survival curves of the high and low-risk group of the TCGA test set. (d) Time-dependent ROC curves of FAM83D-associated prognostic model of the TCGA test set

**Figure 10.**
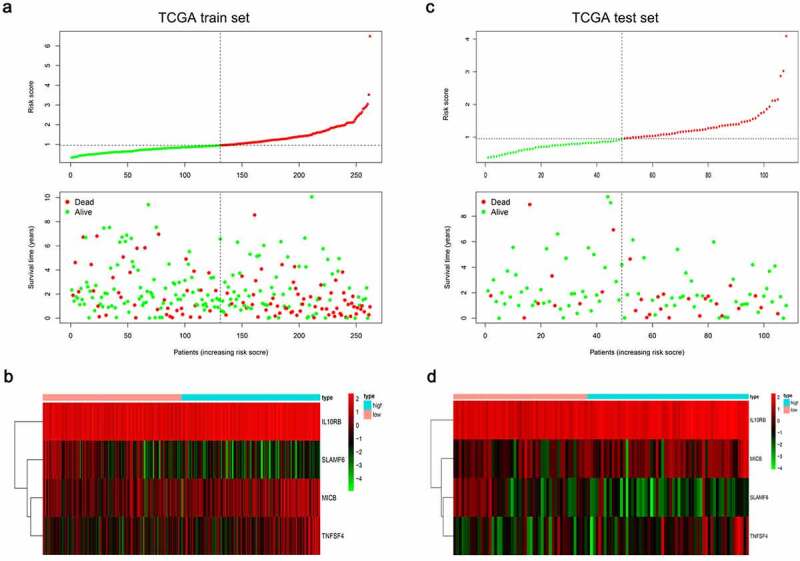
Characteristics of the FAM83D-associated prognostic signature in the TCGA dataset. The dotted line is the optimal cut-of value for dividing HCC patients into high and low risk groups. (a) The distribution of risk-score and the survival status of HCC patients in train set. (b) Heatmap of the FAM83D-associated prognostic signature expression profiles between the high and low-risk groups in train set. (c) The distribution of risk-score and the survival status of HCC patients in test set. (d) Heatmap of the FAM83D-associated prognostic signature expression profiles between the high and low-risk groups in test set

**Figure 11.**
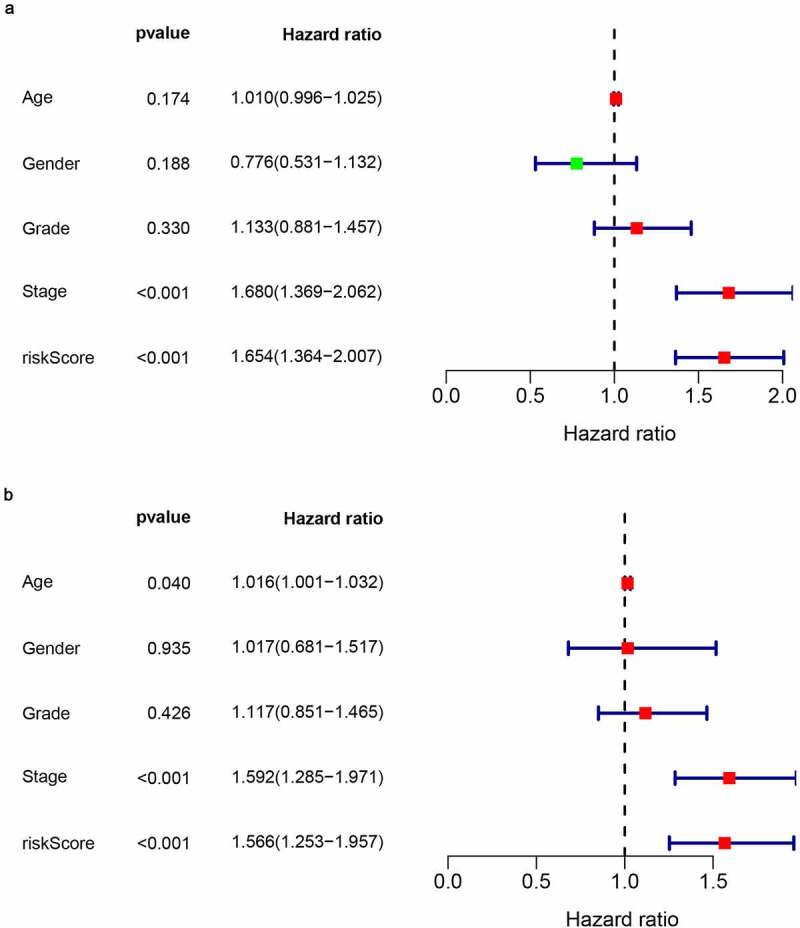
The prognostic value of the FAM83D-associated prognostic signature in the TCGA dataset. (a) Univariate COX analysis of risk-score and clinical characteristics. (b) Multivariate COX analysis of risk-score and clinical characteristics

In order to assess the prognostic value of 82 FAM83D-related immunomodulators, we firstly identified 4 genes (SLAMF6, IL10RB, MICB and TNFSF4) in train set by univariate COX analysis that were significantly related to the prognosis of HCC (Figure 8a). Then multivariate COX analysis identified these four genes as the prognostic signature in HCC (Figure 8b). The risk-score was calculated by accumulating the product of these 4 genes expression and its coefficient in each sample. The Kaplan Meier survival analysis revealed that the low-risk patients had a relatively longer OS than the high-risk patients did (Figure 9a). The area under the ROC curve for the 1-year, 3-year, and 5-year survival probabilities were 0.709, 0.688 and 0.657, respectively (Figure 9b). The distribution of risk-scores, survival status and characteristic gene expression pattern of HCC was shown in Figure 10a-b. The results were verified in the test set (Figure 9c-d and Figure 10c-d). Univariate COX regression analysis demonstrated that the risk-score as well as tumor stage were associated with OS (Figure 11a), and the multivariate COX showed that the risk-score was an independent risk factor for the prognosis of HCC patients (Figure 11b).

### 3.7 Construction of nomogram

**Figure 12.**
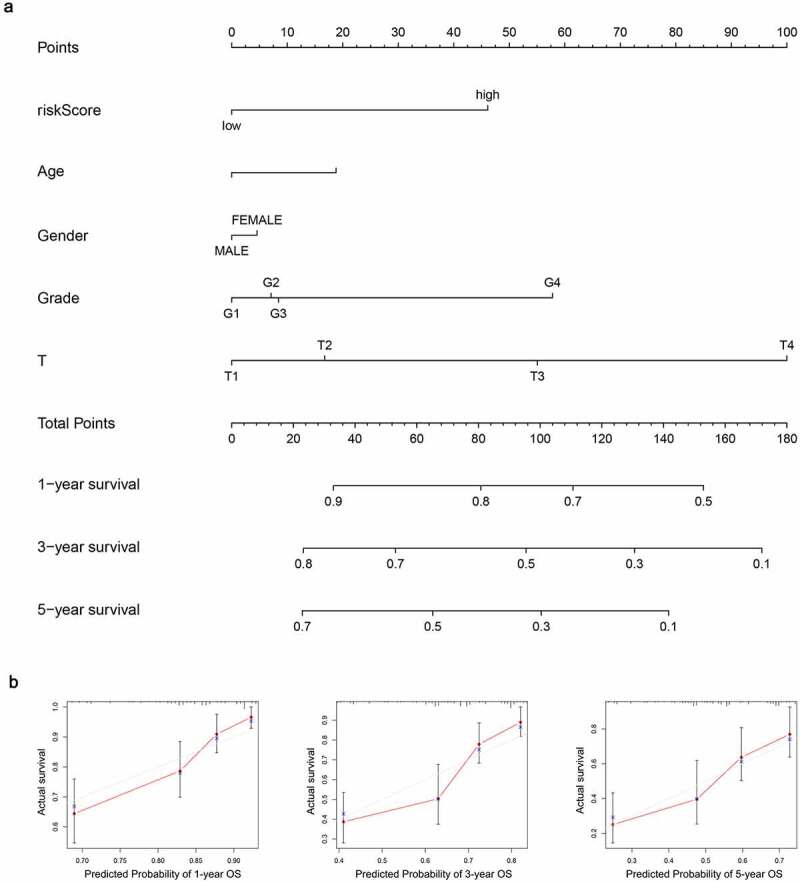
Establishment of the prognostic nomogram with the inclusion of the risk-score. (a) A nomogram for predicting 1-, 3- and 5-year survival possibilities of individual HCC patients. (b) The calibration curve of 1-, 3- and 5-year survival of HCC patients. The dashed line represented a perfect uniformity between nomogram-predicted and real possibilities.< 0.05

A nomogram was created to anticipate the survival probability of HCC patient (Figure 12a). The curve of calibration indicated a good fit between nomogram-predicted probability and idea reference line for the 1-, 3- and 5-year survival (Figure 12b). Additionally, the C-index was 0.66, which suggested a good predictive power.

## 4 Discussion

Tumor immune is a complex procedure including antigen presentation, T cell activation, avoiding local inhibition, re-stimulation by tumor APC, and tumor cell killing [27]. If any of these critical steps is unable, the efficiency of immunotherapies may be jeopardized. Therefore, there are still major challenges in discovering more effective immunotherapy drugs and selecting patients suitable for the treatment. Biomarkers that reflect actual a patient’s immune response and prognosis are remarkably useful for making better HCC treatment decisions.

Many studies have proven that FAM83D plays an important role in liver cancer. Liu et al. reported that the expression levels of FAM83D mRNA in HCC are linked to gender, AJCC stage, overall survival time and disease-free survival time [13], but the mechanism is unclear. Unlike previous research, we found that the immune effect of FAM83D in HCC has not been studied yet. In this study, we focused on the relationship between FAM83D and the immune status of HCC. Tumor micro-environment plays an indispensable function in the pathogenesis of cancers [28,29]. We found that the expression of FAM83D was linked to immune cell infiltration levels and immunomodulaters in HCC. Using stepwise COX regression analysis, we screened four immune-related prognostic genes among FAM83D-related immunomodulators and established a multi-gene prognostic model. Subsequently, the predictive power of the model was internally tested.

Firstly, a comprehensive analysis was conducted on all HCC patients in the TCGA database to calculate the components of immune cell infiltration in their tumors, thereby reflecting the changes in the immune status of HCC patients. The infiltration traits of 22 immune subgroups in HCC have changed significantly compared to normal tissues, according to a CIBERSOT analysis. In addition, some immune cell subgroups were found to be related to HCC OS. Similar immune status for HCC also have been shown in previous studies [8,18]. However, compared to obtaining the patient’s immune cell infiltration characteristics, biomarkers that reflect HCC patients’ immune status was more achievable can be investigated further.

The abundance of most immune cell types was inversely proportional to the levels of FAM83D mRNA via TISIDB network tool. GSEA of TCGA datasets showed that high FAM83D expression regulates several pathways such as mTOR and P53 signaling pathways. Previous studies have determined that PI3K/Akt/mTOR and TP53 signaling pathways are the effective immune therapeutic targets in HCC [30,31]. These results confirmed the link between FAM83D and immunity.

Xu et al. have reported that the expression of Rad51 gene is related to immune infiltration in HCC and could be used as a prognostic biomarker using the Cox regression analysis [32]. In the present study, four FAM83D related prognostic immune genes, including SLAMF6, IL10RB, MICB and TNFSF4 were obtained using the similar methods. SLAMF6 was a prognostic protective factor for HCC patients, while IL10RB, MICB and TNFSF4 were the prognostic risk factors according to our Cox regression analysis. Liu et al. determined that SLAMF6 is a prognostic gene based on calculating the stromal score and immune score in HCC [33]. Ma et al. found that IL10RB polymorphisms have an impact on the progression of HBV-related liver diseases, especially HCC [34]. Cadoux et al. have reported that the expression of NKG2D ligand (MICB) is down-regulated by β-catenin signaling and is associated with HCC invasiveness [35]. Yao et al. have proved that DC-mediated TSLP-OX40L (TNFSF4) pathway is an effective drug target that can improve Th2 immunosuppression in HCC [36]. However, unlike previous studies, we have clarified the close relationship between the four prognostic genes and FAM83D gene, and combined them to further explore the potential mechanism of these genes in HCC and their relationship with immunotherapy using GO and KEGG pathway enrichment analysis.

Liver is unique in that it has a significant impact on immune regulation as well as many metabolic functions [37]. There is an active, immune-mediated inflammatory process in liver cirrhosis [38]. An analysis of KEGG signaling path of FAM83D-related immunomodulators demonstrated that there were several pathways such as NK cell mediated cytotoxicity, NF-κB signaling pathway and T cell receptor signaling pathway could be engaged in the FAM83D-mediated immune response.

T cells, which are immune cells, regulate most immune system processes. T cells could recognize tumor antigens in the tumor micro-environment, which antigen-presenting cells deliver to T cell receptors (APCs). In patients with HCC, the number of normal T cells decreased while the number of damaged T cells increased, resulting in cancer progression [39]. Sorafenib, a multi-target kinase inhibitor, activated cytotoxic NK cells, resulting in tumor cell death [40]. NF-κB is involved in the initiation and progression of HCC as well as fibrogenesis in chronically inflamed liver [41]. The findings show that immune-related pathways regulated by FAM83D-related immunomodulators have positive effects in HCC.

In light of the fact that there are very few studies on FAM83D’s direct interaction with the three pathways mentioned above. FAM83D, on the other hand, has been reported to be associated with regulating the AKT pathway and mediating tumor proliferation [42,43]. In addition, the AKT pathway has been implicated in the regulation of NK cells, T cells, and the NF-B signaling pathway in the tumor immunity process [44–46]. Combined with our findings, FAM83D inhibitors are biologically feasible, even though the specific mechanism of FAM83D regulating immunity has not been experimentally confirmed.

Finally, the immune prognosis model for HCC via FAM83D-related immunomodulators was constructed in this study. Risk-scores extracted from the four gene signature were found to be tightly related to HCC survival in this model. A nomogram was created for individualized survival anticipation, with a C-index of 0.66. These results were verified by internal validation.

With the analysis of public data, we found the relationship between FAM83D and HCC immune micro-environment. Hope in the future, experimental study can verify these findings further.

## 5 Conclusion

FAM83D may involve in the regulation process of the HCC immune micro-environment and present a poor prognosis. SLAMF6, IL10RB, MICB and TNFSF4 as the key prognostic immune genes these were associated with immune micro-environment and the prognosis of HCC. The prognosis predicted model generated by the four FAM83D-related immunomodulators could be adopted to anticipate the prognosis of HCC patients. However, the larger cohorts are needed for external study to verify the results of this study.

## Supplementary Material

Supplemental Material

## Data Availability

Publicly available datasets were analyzed in this study, these can be found in the Cancer Genome Atlas (https://portal.gdc.cancer.gov/).

## Highlights

1. FAM83D would be a prognostic immune gene in HCC.
2. SLAMF6, IL10RB, MICB and TNFSF4 were identified as FAM83D-reated prognostic immune genes.
3. High expression of SLAMF6 might relate to a better prognosis for patients with HCC.
