# Supplementary figures and images for: Immune implication of FAM83D gene in hepatocellular carcinoma
Source: Bioengineered. 2021 Jul 24;12(1):3578–92. doi: 10.1080/21655979.2021.1950260 (PMC8806426; doi:10.1080/21655979.2021.1950260)

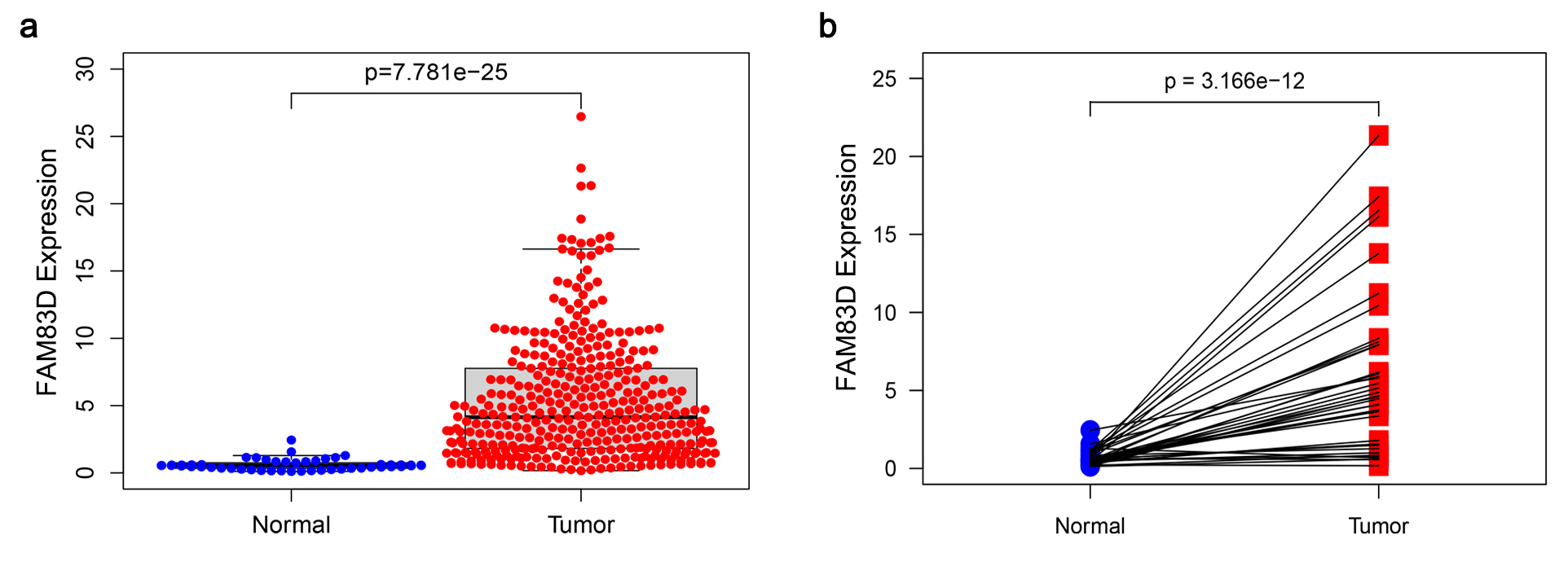

Supplement: Supplemental Material [file KBIE_A_1950260_SM8097.zip › supplementary/Supplementary Figure 1.jpg]

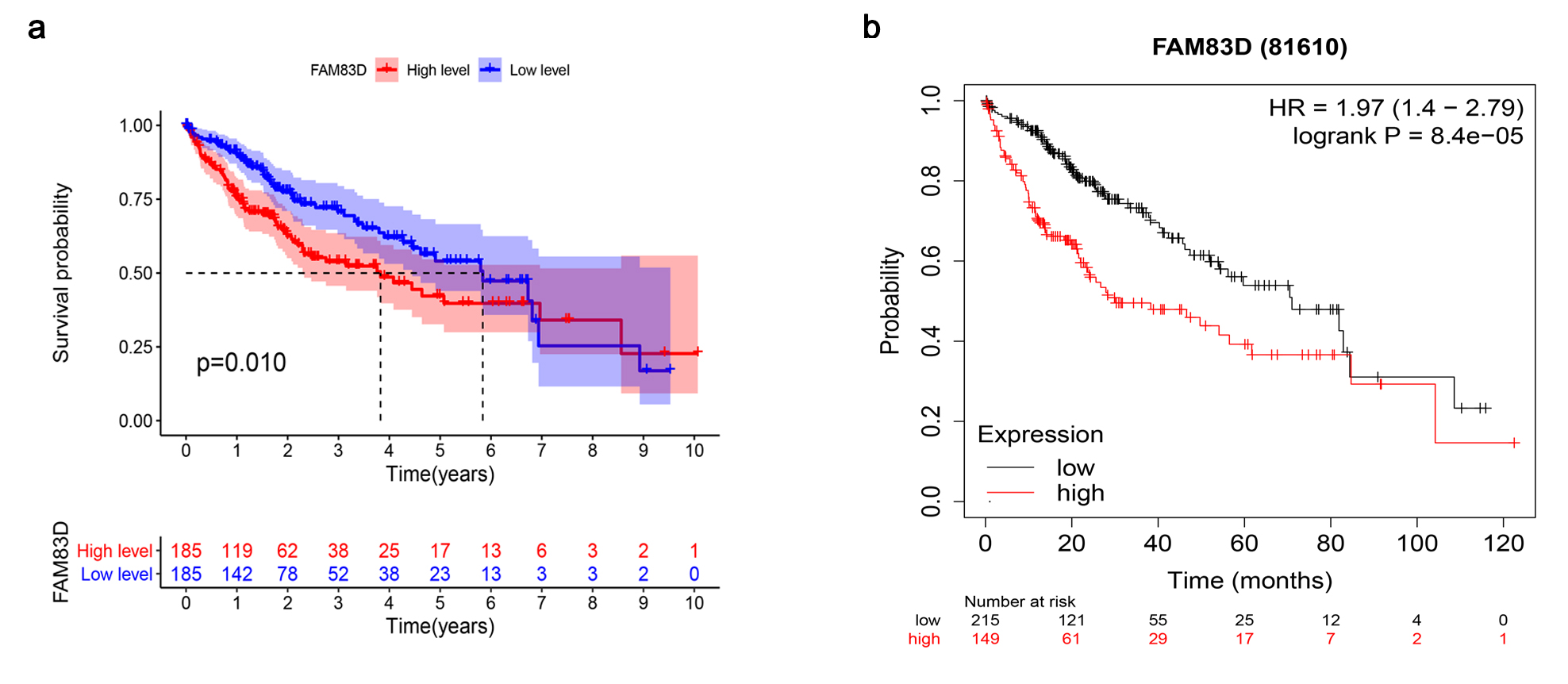

Supplement: Supplemental Material [file KBIE_A_1950260_SM8097.zip › supplementary/Supplementary Figure 2.jpg]
